# Co-producing an online patient public community research hub: a qualitative study exploring the perspectives of national institute for health research (NIHR) research champions in England

**DOI:** 10.1186/s40900-024-00556-4

**Published:** 2024-02-16

**Authors:** Eleanor Hoverd, Sophie Staniszewska, Jeremy Dale, Rachel Spencer, Anne Devrell, Dena Khan, Carrol Lamouline, Sanya Saleem, Pam Smith

**Affiliations:** 1 Academic Primary Care, Warwick Medical School, Warwick University, Gibbet Hill Rd, Coventry, CV4 7AL England; 2 Warwick Medical School, Warwick Research in Nursing, Warwick University, Coventry, England; 3 University of Central London, London, England

**Keywords:** Patient public involvement and engagement (PPIE), Patient public involvement (PPI), Digital resource, Higher educational institutes, Research involvement

## Abstract

**Background:**

Patient and Public Involvement and Engagement (PPIE) should be embedded as part of researchers’ everyday practice. However, this can be challenging. Creating a digital presence for PPIE as part of Higher Education Institutes’ (HEIs) infrastructure may be one way of supporting this. This can support how information is made available to patients and members of the public, but relatively little is known about how HEIs can best do this. Our aim was to develop a university website for patients and members of the public to learn about ways to get actively involved in research and be able to access the results of health and social care research.

**Methods:**

This project involved working as partners with five National Institute for Health and Care Research (NIHR) Research Champions. NIHR Research Champions are volunteers who raise awareness and share experiences about health and social care research. Content of a prototype *Patient Public Community Research Hub* website was co-produced with the Research Champions, and then 15 NIHR Research Champions from across England were asked for their views about the website.

**Findings:**

The information collected told us that the *Patient Public Community Research Hub* was viewed as being beneficial for increasing visibility of PPIE opportunities and sharing the findings of studies though needs further work: to make the information more user-friendly; to improve the methods for directing people to the site and to create new ways of connecting with people. It provides a foundation for further co-development and evaluation. A set of recommendations has been developed that may be of benefit to other HEIs and organisations who are committed to working with patients and members of the public.

**Supplementary Information:**

The online version contains supplementary material available at 10.1186/s40900-024-00556-4.

## Plain language summary

Sharing the results of health and care research studies with patients and members of the public could be improved. In many cases, patients and members of the public do not receive the results of studies they have taken part in. As well, it should also be easy for patients and members of the public to find out about opportunities to get involved with researchers in the development of their research. Universities have an important role to play in providing opportunities for patients and members of the public to be involved in the development of research studies, as well as sharing the findings of their studies. Creating an online patient public community research hub for this purpose was co-produced with National Institute for Health and Care Research (NIHR) Research Champions. The aims of this research were to find out what research volunteers within the National Institute for Health Research (NIHR), in the UK, would like to find on a university website about health and care research. This research aimed to understand how best to raise awareness about how people can get involved in research. It also aimed to understand how best to share information about research, with patients and members of the public, from a university website. Five NIHR Research Champions from diverse ethnic and cultural backgrounds (including younger and older people) helped to develop a set of webpages on a university website, called the Patient Public Community Research Hub. Once the initial online hub was created, online interviews were held with another 15 NIHR Research Champions. The interviews were to help the researcher to understand what they thought about the Patient Public Community Research Hub. The results from the interviews were analysed and grouped into themes. The themes helped to tell us what NIHR Research Champions felt patients and members of the public would want to see on the Patient Public Community Research Hub and what areas needed improving. A co-produced set of recommendations was created with the NIHR Research Champions who helped to shape the Patient Public Community Research Hub. The recommendations are for researchers, other organisations, or services to use. These recommendations along with the findings may help to improve how information gets shared about the results of research and ways in which patients and members of the public can get involved.

**Supplementary Information:**

The online version contains supplementary material available at 10.1186/s40900-024-00556-4.

## Background

Higher Educational Institutes (HEIs) have a crucial role to play in fostering Patient Public Involvement and Engagement (PPIE) in health and social care research [1–4]. This is needed at institutional, departmental, project and individual researcher levels to ensure PPIE is embedded at this organisational level of the health and care research system and the results of health and care research results are made publicly available [5]. PPIE encompasses raising awareness of, and enabling involvement in health and social care research[6]. Ensuring PPIE is rooted in academic practice and within the infrastructure of HEIs is known to be challenging [6]. Creating ways to support an infrastructure that incorporates PPIE within HEIs, through constructing space for improving knowledge about health and social care research, as well as opportunities for becoming involved may improve engagement[7]. Evidence is lacking about how HEIs’ websites can support this process. Better understanding is required of what patients and members of the public want and need [8].

The UK policy paper ‘Saving and Improving Lives: The Future of UK Clinical research delivery’ (Gov.uk 2021) commits to creating a patient-centred research environment, making it easy to participate in health and social care research studies. Ideally, this should include creating a patient-centred online research environment. The National Institute for Health and Care Research (NIHR) Centre for Engagement and Dissemination (CED) has worked with public contributors to develop an improvement plan focussing on partnership working, based upon the original priorities set out in the ‘Going the Extra Mile’ report[9]. ‘Going the Extra Mile’ was a review of patient and public involvement in research in the NIHR, which is the main funding body for health and social care research in the UK, setting out a clear goal that by 2025 all patients and members of the public should be aware of and have the choice to take part in health and social care research [9]. One of the five key areas for improvement identified was around online engagement.

It is important to understand how patients and members of the public may access involvement and engagement opportunities about health and social care research online [10, 11]. Evidence in the online communications field suggest that websites should be developed according to the unique needs of those they wish to engage with. It is unclear how successful current methods have been [12–14].

In the project described here, we worked with a stakeholder group of NIHR Research Champions who are volunteers with experience of Patient Public Involvement Engagement (PPIE) to develop the initial content of an online community research hub embedded within Warwick Medical School’s Unit of Academic Primary Care webpages [15]. The *Patient Public Community Research Hub* can be found at https://warwick.ac.uk/ppch. It seeks to promote PPIE opportunities and to improve accessibility to the findings of studies completed by this unit [16]. We then sought to evaluate what NIHR Research Champions felt ought to be part of the Patient Public Community Research Hub and whether it met their needs.

This study aimed to answer the following questions:

1. What would NIHR Research Champions like to find on the Patient Public Community Research Hub?
2. How does the Patient Public Community Research Hub raise awareness about PPIE opportunities and share information about health and social care research, to meet the needs of users?

The findings were intended to provide deeper insight into what is important to include in an online *Patient Public Community Research Hub* for sharing information about research [14, 17, 18], and to generate recommendations for other HEIs, or research organisations who may host such hubs.

## Methods

### Initial development of the hub

A co-production group of five NIHR Research Champions met virtually via Microsoft Teams on four occasions (NIHR 2023). NIHR Research Champions are individuals who may be patients, may have taken part in a health and social care research study before, and have some understanding of the health and social care research process (NIHR 2023). They are involved in various research processes including: reviewing and developing research materials, providing feedback on research grant applications, developing research questions and projects with researchers, promoting research opportunities to members of the public.

The NIHR Research Champions met for hour-long meetings, approximately every six weeks, from the beginning of September 2020 over a period of six months to co-create the initial content of the *Patient Public Community Research Hub* webpages. The group represented older and younger adults, with three people from diverse ethnic and cultural backgrounds. Co-production is a way of working in equal partnership with patients and the public, sharing responsibility and power [19]. Through a series of virtual discussions, the group identified topics that were felt to be important to include on the webpages. This included information about local studies, local researchers, general research information, PPI opportunities, patient stories and information on any events. A brief scoping exercise that entailed looking at information provided about PPI in health and care research locally, was undertaken as a starting point, to consider what the basic requirements may be for building a *Patient Public Community Research Hub*. The top six HEIs who achieved excellence in quality of health and care research according to the most recent Research Excellence Framework (REF) results were selected for the exercise [20]. The information on these HEI webpages provided general information about PPI, with links to newsletters and local opportunities which helped to gain an understanding of the type of information being shared. None were found to share findings of local research in Plain Language.

An initial prototype was created, allowing the group to be “walked” through the layout and information. Further amendments were made before agreeing that the webpages were ready to be tested through interviews with a wider group of NIHR Research Champions. This approach enabled the NIHR Research Champions to shape the hub from the outset [19]. Details of their input is described further under the analysis sub-section. Members of the group were reimbursed for their time as per NIHR CED guidance [21].

### Design and setting

Study interviews and meetings were held virtually, and recorded via Microsoft Teams, as discussions with the co-production group and recruitment began during the COVID-19 pandemic when social distancing was required. A qualitative approach was used with ‘think aloud’ interviews, followed by semi-structured interviews one week later [22]. Think aloud interviews allow the participant to share their thoughts about their experience and reactions when undertaking a set of tasks, in their own environment [23]. It is a method that helps to identify usability issues [23]. This information was built upon using semi-structured interviews as a follow-up method [24]. A topic guide created a structure for the interview discussions, through enabling some flexibility for spontaneity to gather a richer understanding of each individual’s thoughts, feelings and perceptions about an area in which they have some knowledge, or previous experience [25]. Appendix A presents the topic guide used for semi-structured interviews. The study was approved by University of Warwick’s Biomedical Scientific Ethics Committee (BSREC 151/19-20).

### Participants and recruitment

Once the co-development of the prototype of the hub had been completed, 15 NIHR Research Champions were recruited via invitation through the Clinical Research Network (CRN) West Midlands Patient Public Involvement and Engagement (PPIE) leads and via a national website *peopleinresearch.com* [26]. The CRN West Midlands is one of fifteen Local CRNs supporting delivery of health and social care research in coordination with the NIHR [27]. PPIE leads are employed by the CRN West Midlands as research delivery staff who co-ordinate PPIE activities and groups, including NIHR Research Champions. Two PPIE leads were contacted via email with a participant information leaflet, invitation letter and sample consent form to share with their respective PPIE groups of NIHR Research Champions.

All potentially eligible participants who expressed an interest, consented to taking part following discussion of the project and informed consent according to Good Clinical Practice (GCP) guidelines [28]. Each NIHR Research Champion was interviewed twice, with no participants dropping out.

### Data collection

Think aloud interviews lasted no longer than 30 min and enabled participants to ‘walk through’ the webpages of an early prototype of the *Patient Public Community Research Hub*, to share their thoughts, attitudes and perceptions about the proposed online community research hub. These were followed one week later, by a semi-structured interview lasting up to an hour, to better understand their views and priorities.

### Analysis

Each interview was transcribed verbatim using Microsoft Teams transcription in addition to playing back recordings to correct any anomalies. Analysis was conducted using framework analysis with themes and sub-themes created deductively from the areas of interest articulated by participants and literature review, and inductively where new themes and sub-themes were identified from the transcripts and in discussion with the co-production group [29]. All authors met to discuss the thematic categories and the co-production group validated final themes through discussion. All quotes are anonymised.

## Results

Participants in the study included a diverse range of individuals from the age of 16 years through to early elderly, all of whom had experience of working with health and social care researchers as NIHR Research Champions in England. Six of the interviewees represented people from diverse ethnic and cultural backgrounds in the UK, two of them were young persons from the CRN West Midlands Young Persons Advisory Group (YPAG).

Overall, NIHR Research Champions felt that having a local platform like the Patient Public Community Research Hub would be beneficial to patients and members of the public, though it was important that any local platforms, connect and signpost to national platforms too e.g., *Be Part of Research*. NIHR Research Champions believed that opportunities to be involved in health and social care research are not visible to patients and members of the public. Presenting these opportunities at a local level may help to embed PPIE within university infrastructures and increase visibility. It may also help to demonstrate to patients and members of the public why PPIE is necessary, how individuals are involved, the impact their involvement has had and what the findings of the research were, which NIHR Research Champions report as not always being fed-back. Four core themes were identified as being important to an online *Patient Public Community Research Hub:* content; usability; signposting and connectedness (Table 1). These themes indicated what was acceptable, as well as areas that required further improvement, with real-world examples provided in the following sections.

**Table 1:** Thematic structure

Theme	Subthemes	Examples
Content	Research literacy	Researchers and universities can seem intimidating
	Empowerment	Wider lack of awareness amongst public of how to get involved in research
	Interaction	Move away from traditional methods of dissemination
	Communicating about PPIE	Frustration about “not hearing back” if you have helped out
	Information about studies	Dislike of term “lay summaries”
	Dissemination of findings	Frustration with not seeing how research has changed practice for patients
Usability	Too academic Corporate Less is more Visuals	Elitist Hard work, cognitively demanding to work through webpages (information overload) Want to connect and talk to researchers Can feel overwhelming with too much information Navigation was tricky for some with some confusion over what things were in each section and occasionally inadvertently veering off the hub to other pages Unclear at times as to who sections were intentionally for
Signposting	Links via trusted organisations, other groups Visibility on noticeboards, social media Tailor messages/information to specific groups	Suspicion over websites, sites text message that are not associated with familiar organisations e.g., NHS Traditional methods e.g., community noticeboards are still acceptable for sharing information Some organisations/ groups e.g., Patient Panel Groups within General Practices may not know that they could help to signpost, and how
Connectedness	Equality, Diversity, Inclusion Disconnect Connections to researchers and universities Active partnership with HEI	Sense of belonging Not being heard Not feeling connected Feeling that macro level organisations are not connected to individuals Research may be viewed as an “academic exercise” not based on real need

### Theme 1: content

Think aloud and semi-structured interviews revealed that participants felt that content on the *Patient Public Community Research Hub* should include the local health and social care research projects and their findings, general information about health and social care research, opportunities to get involved and information about events. Participants identified three key aspects that they thought were important to consider when further developing the content of a *Patient Public Community Research Hub*: the use of research and technical language, preferences for what should be included and limiting the amount of written text.

### Research language

Participants felt that information about health and social care research displayed on the hub may not be well understood by the general public. Some felt that people would need to be ‘research literate’ to understand general information about research because information about research can sometimes be full of jargon and scientific terms that can hinder communication and understanding [30]. This was seen as a major barrier to the public accessing information from the hub. While the participating NIHR Research Champions felt that they usually understand ‘research language’, but other people who are not regularly involved may not have that same understanding.

‘*If people can understand the language being used, they will be more likely to feel like they can be part of research.*’ Participant 2, Think Aloud Interview

Many felt that information about health and social care research itself including Plain Language Summaries that were on the hub were generally too complex for most people to understand.

‘*You talk about the NIHR which obviously somebody like myself has heard of, but if I knew nothing about research, it would tell me nothing*.’ Participant 5, Think Aloud Interview

### Preferences for content

Participants felt that there is a wide lack of awareness amongst the public about how they can be involved in research, with a suggestion that more talks or webinars by researchers with the public are needed. Some felt that a move away from more traditional methods of dissemination such as Plain Language Summaries (PLS) to more creative ways of sharing the findings of health and social care research was needed, with a preference for visual information, such as videos and infographics.

*‘It can go above and beyond just lay summaries. I’m always keen to think about other ways for dissemination and whether this is the right place to start thinking about, you know, like performing arts again as a kind of visual medium to disseminate research results. It is something that I think is quite underused and could be used a lot more. Or you know, even things like sort of photography or art*.’ Participant 7, Semi-structured Interview

Some participants also spoke of their dislike for the term “lay summaries”.

‘*I’ll be honest, I don’t like the term lay summaries, but that’s just personal. It’s widely used. Yeah, I just find it personally a little condescending*.’ Participant 8, Semi-structured Interview

General information like newsletters about local health and social care research studies are valued and should be easily located as part of the *Patient Public Community Research Hub*. Several participants suggested that the hub should provide a link to a glossary of terms as a helpful reference, to improve understanding ‘research language’. Some also suggested that they would like to see the hub share opportunities to talk about potential research ideas with health and social care researchers at universities.

### Theme 2: usability

Usability was about creating a user-friendly interface to improve accessibility to PPIE opportunities and increase transparency of findings. Usability focussed on how participants navigated the hub and its audio-visual aspects.

The use of colour and brighter images was felt to be more attractive, especially to younger audiences, with an opportunity to interact in an enjoyable way. The lack of bright images and colour was felt to contribute to an un-inviting, academic feel.

‘*It needs to be more visual, and it needs to be direct if you’re trying to maybe engage with the public and of different ages, especially younger ages, might appreciate some brighter colours*.’ Participant 5, Think Aloud Interview

There was a strong preference for a more informal, visual look to the webpages.

‘*It looks like it’s going to be hard work to read through and find something of relevance to you, whereas if they were little pics and icons with the links to give you some kind of inkling what they’re about*.’ Participant 4, Think Aloud Interview.

‘*It’s a well-known saying a picture paints 1000 words, but it does, in terms of cognitive accessibility and reducing cognitive load*.’ Participant 6, Semi-structured Interview

### Theme 3: signposting

Signposting refers to the way in which patients and the public are directed to local and national opportunities about PPIE. Participants felt this is best done by maximising visibility through existing, trustworthy sites e.g., charities. Many reported suspicion over websites, sites, text messages that are not associated with familiar organisations such as the NHS. In addition, providing opportunities for learning and engaging with researchers was felt to be an important element in encouraging people to visit the *Patient Public Community Research Hub.*

‘*If there could be some sort of collaboration or link with the NHS so that people can get directed to it from an existing NHS web infrastructure, which might be quite good*.’ Participant 14, Semi-structured Interview

Participants felt that offering training opportunities and live webinars would encourage people to visit the site, as well as ensuring that it is refreshed with new information based upon feedback from visitors to the site. In order to encourage people to return to the Patient Public Community Research Hub, it was suggested that regular updates through joining a mailing list may be helpful.

Participants questioned how under-served communities such as individuals from diverse ethnic and cultural backgrounds, or homeless people may find, and be included in the hub, suggesting that it is important to consider the local context of a population.

‘*I think trying to broaden engagement amongst the wider population is a challenge definitely, so I think straight away you could potentially be excluding by having an online space. People who aren’t confident using it, don’t want to use it, can’t afford to use it..uh, online information is not easily accessed for members of the traveller community. There are a lot of different groups that can’t access online information so straightaway there is a potential exclusion*.’ Participant 8, Semi-structured Interview

### Theme 4: connectedness

Connectedness emerged as an important theme around wanting to connect with researchers and how this may be facilitated by the hub. Developing a means of connection with members of the public through the webpages of the hub was viewed as one of the most important areas to address with challenges around digital poverty, cynicism around the internet and the negative impact that online communication can sometimes create.

### Belonging

Some participants highlighted the importance of belonging and what it feels like to be involved with health and social care research. Some participants expressed how this may feel in terms of sharing ownership.

‘*It’s like *joining* a family*. ‘Participant 1, Semi-structured Interview

‘*The patients feel like they are part of the team’* Participant 15, Semi-structured Interview

It was viewed as important to be able to see people’s faces on webpages about health and social care research, adding to the approachability of the research. Images of people should feature on the landing page to the hub.

*‘The first thing you see is a friendly face.*’ Participant 12, Think aloud Interview

Participants also liked seeing photographs of other patients and members of the public, accompanied by their stories.

One participant highlighted that establishing relationships that consider the needs of patients and members of the public is an important aspect of engagement.

‘*It’s about respectful relationships well ahead of the research*.’ Participant 5, Semi-structured Interview

## Discussion

This study provides valuable understanding about what is likely to be important in developing a virtual *Patient Public Community Research Hub,* and highlights the importance of carefully considering the structure, content and format of information included in order that it is useable and of interest to patients and members of the public. There was some dislike of the term “lay summary” and a preference for findings of studies to be shared in more interactive formats. Images and videos may help engagement and creating an accessible feel. Linking the *Patient Public Community Research Hub* from other trusted sites e.g., NHS, or charities, was suggested as being a potential way to signpost people to it. Generally, it was viewed that an online space like this is valuable to patients and members of the public but requires iterative development to keep it updated and engaging.

The information on the webpages was initially determined by a co-production group of NIHR Research Champions with the intention of further co-creation and development based upon the findings of the study. Key recommendations based upon what the participating NIHR Research Champions perceived as being most important are shown in Table 2.

**Table 2:** Hub-specific recommendations

Area	Recommendations
Content	Use more infographics and minimal text on webpages Plain Language Summaries should be co-written with patients and members of the public and text kept to a minimum Provide links about health and care research with more detailed information, behind simple icons, or images Use more creative methods for sharing information with patients and members of the public Newsletters are valued sources of information about local health and care research projects and opportunities A link to a glossary of research terms would be helpful Avoid using the term ‘lay summaries’ as it is viewed as condescending. Researchers should use the term Plain Language/ English summary, as this is preferable
Usability	Ensure navigation is easy through providing “back” buttons and “search boxes” Ensure colour combinations are carefully selected Use audio and subtitles on videos An informal feel is preferred so as not to feel “too academic” The option to provide comments or provide feedback would be valued
Signposting	Use trustworthy sites e.g., via NHS platforms, Patient Panel Groups associated with General Practices, charities, support groups, or organisations to help to signpost to the hub Signpost to hub in non-digital ways e.g. community noticeboards Advertise on social media to reach under-served communities
Connecting	More photos of health and social care researchers on webpages would create a more approachable feel Online PPIE events with researchers, led by members of the public, would help to develop relationships between patients, members of the public and HEI’s Patients and members of the public would like more interaction with researchers Interactive functions to share thoughts and experiences of health and care research would be valued

### Further development of an online community research hub

The four key themes identified: content, usability, signposting and connectedness may guide reflection of further development of online community research hubs and will be discussed.

In addressing content, there needs to be a clear understanding of who the hub is for, what it is for and how it can be used. The hub needs to provide information relevant to a local context as well as additional, more general information about health and social care research in a way that is visually attractive, interactive and uses minimal text. Images and bright colours may have the potential to enable engagement and involvement as they are important elements to stories, which can make scientific research more engaging [31].

The second point relates to usability. It is critical for online web platforms to be easy to use, memorable, engaging and should invoke positive feelings in the user [32]. NIHR Research Champions also indicated that ensuring usability is inclusive is critical. More needs to be done to tailor a hub around the specific requirements of different communities [33]. It is recognised that online resources may exclude some groups and therefore, should not be the only method used to engage with patients and members of the public, although there are ways to adapt and make platforms more flexible for a wider range of users [13, 32].

Colour palettes should be carefully considered to ensure they do not reduce accessibility to the webpages [34]. Sensitivity to cultural differences such as the meaning behind different colours, gestures and formatting should be explored in further research with a more diverse group of users [32]. It is very important for users who are less confident with digital technology, or have limited educational backgrounds, that information is clear and kept to a minimal, to help with attention and understanding [34]. Providing audio with subtitles, ensuring interfaces are kept simple with user-tested methods for remembering information can create wider accessibility [32].

Thirdly, signposting to the *Patient Public Community Research Hub* between, and from, trusted organisations, was important with participants reporting that linking the hub via an existing NHS website, or GP practices would provide credence to the site. Using clear, consistent images that represent trusted health care organisations; e.g., the NHS logo is known to reduce anxiety and stress by patients and members of the public, as it prevents confusion and provides reassurance that a service is genuine and more importantly, is there to look after them [35, 36].

Finally, the need for HEIs to engage with communities which focusses more on building trust and mutual benefit is growing. For example, the Health Determinants Research Collaboration (HDRC) Coventry, England, is building a research infrastructure, working with people in Coventry to better understand their needs and health priorities [37]. However, there is a limit with what has been achieved to date related to a lack of infrastructure and a lack of perceived value in investing in building relationships with communities [38, 39]. Creating a sense of belonging was important to NIHR Research Champions in this study and it is critical to think of solutions to improving the relationship of HEIs with patients and members of the public and the role of hubs in doing this. An example of creating connectedness with communities is from the work of True et al. (2021) who report ‘*institutions don’t hug’* but through using photographs and stories to share findings of research this can lead to social connectedness which was especially true in their study with US Veterans – a community that has had negative experiences of healthcare [39]. From an online perspective, using images that include human faces has been shown to increase engagement on social media platforms such as Instagram, presenting an opportunity to HEIs to showcase its researchers as “*warm, relatable human beings*”[40].However, it is potentially challenging to consider how to increase connectedness between patients, members of the public and health and care researchers online, as the needs and wants of consumers are continuously evolving, so online approaches to building relationships must keep the pace [41].Despite the potential challenges, improving connectedness has been shown to improve trust, an attribute of connectedness [42].

Trust is a crucial aspect of health and care research nurtured through positive relationships and transparency of information communicated to participants in studies, as well as patients and members of the public [43]. The UK’s Health Research Authority “*We make it easy to do research that people can trust strategy 2022–2023*” encourages researchers, research departments and institutions to make it easier for patients and members of the public to become involved in research and share their findings. A *Patient Public Community Research Hub* provides a mechanism for doing so at a unit, or department level. Researchers and research institutions must demonstrate their trustworthiness, which is particularly important for building relationships with under-served communities [44]. Creating a web-platform to improve connectedness with patients and members of the public could support this strategy, though further research is needed to understand how this may impact on trust.

Increasing numbers of HEIs have dedicated online spaces with information about Patient Public Involvement and Engagement (PPIE) though there is further scope to develop these spaces to be more useful and engaging for patients and the public. However, additional resource to build and maintain such platforms is likely needed. The findings of our study will help to shape current, digital platforms that aim to raise awareness about opportunities for involvement and engagement in health and social care, as well as contributing to advice for researchers about how to best share information of findings. However, this core issue around connectedness warrants further exploration with a diverse range of populations to understand what it means to patients and the public to be connected with HEIs and health and care researchers.

## Strengths and limitations

A strength of this study is that it was co-produced with NIHR Research Champions who brought a wide range of experience, prior knowledge and understanding of health and social care research. Some brought experience of being part of a Young Persons Advisory Group at a Children’s hospital, whilst others had the experience of working with Clinical Research Networks to raise awareness about studies, some have been co-applicants, involved in reviewing and developing research materials for studies and one individual has experience of chairing a large network of NIHR Research Champions working closely with senior leaders in a research delivery organisation. This strength, however, is also a limitation in that this group are more research aware and interested than most patients and members of the public. In addition, the group were a mixture of previous professionals and university students (one a PhD student) and so were unrepresentative of the wider population. In addition, there was a small sample size though it was not designed to achieve saturation across the diverse range of individuals that may use a *Patient Public Community Research Hub.* Future research in this area, co-produced with more specific groups of individuals such as those from Black, Asian or ethnic minority communities, or individuals with learning difficulties would improve understanding of the requirements necessary to ensure a webspace such as this is inclusive. However, there were also methodological strengths of this study through using think aloud interviews followed by semi-structured interviews to consider how a hub may work for patients and members of the public. These methods also meant deeper insight could be sought into how members of the public may approach, engage with and use a hub like this. The study was conducted at one site though may be applicable to other HEIs and health and care organisations wishing to engage with patients and members of the public.

## Conclusion

The *Patient Public Community Research Hub* offers a starting point for future research around the benefits of engaging with patients and the public in an online environment for health and care research. The findings from this study may aid in the development of future patient and public websites, particularly for researchers who need to build their own websites for studies or for HEIs and organisations developing websites for this purpose. Further evaluation of the usability and usefulness of such platforms would advance understanding about their use (Additional file 1 and 2).

To produce a resource that is likely to be more widely accessed and used, further modification to the content, methods for signposting and more creative approaches for connecting with people through further co-development and evaluation are required. An online community research hub needs to be engaging and needs to bring the human-side of research to its pages through including more images, stories, interactive elements and opportunities to speak to health and care researchers. Other HEIs and research organisations developing online PPIE sites may benefit from considering the recommendations proposed through this project.

### Supplementary Information

**Additional file 1.** GRIPP 2 Reporting Checklist.

**Additional file 2.** Plain Language Summary.

## Data Availability

The datasets used and analysed during the current study are available from the corresponding author on reasonable request.

## Appendix A: indicative Guide for semi-structured, individual interview

**Introduction:** Thank you for taking part in the think aloud interview for the online community space on the Unit of Academic Primary Care webpage last week. This space is intended to be an area where local researchers can share opportunities for members of the public to find out about getting involved and engaging in health research. This might include opportunities to comment on the design of health and care research studies, or even be involved in co-producing research. There may be opportunities to participate in health research studies. The online space also aims to provide lay summaries of local health research findings. Following on from your experience of navigating around the online community space, I would like to ask you some questions to get a better understanding of how you found it.

1. What are your views on the online community space generally? Prompts: Tell me more Why do you think that what might help with that issue?
2. Who do you think will use this online community space to find out about health research, and why would they do that?
3. What do you like best about the site’s appearance and format, what would you change?
4. Are there things you find less helpful about the online community space?
5. What do you think would be the best ways to raise people’s awareness of this community space, and to attract them to look at it?
6. How might this online community space help you or your community in other ways? How else could we use it?

**Final** Are there any other comments you would like to make?

## Abbreviations

CED: Centre for engagement and dissemination
CRN: Clinical research network
HEI: Higher educational institute
HRA: Health research authority
NIHR: National institute for health and care research
PPIE: Patient public involvement and engagement
PPI: Patient public involvement
