# Supplementary material for: Co-producing an online patient public community research hub: a qualitative study exploring the perspectives of national institute for health research (NIHR) research champions in England
Source: Res Involv Engagem. 2024 Feb 16;10:26. doi: 10.1186/s40900-024-00556-4 (PMC10874083; doi:10.1186/s40900-024-00556-4)
Supplement: Supplementary file 1 — Additional file 1. GRIPP 2 Reporting Checklist. [file 40900_2024_556_MOESM1_ESM.docx]

Supplementary materials

From: [GRIPP2 reporting checklists: tools to improve reporting of patient and public involvement in research](https://researchinvolvement.biomedcentral.com/articles/10.1186/s40900-017-0062-2)

| **Section and topic** | **Item** | **Reported on page No** |
| --- | --- | --- |
| 1: Aim | Report the aim of PPI in the study | 5 |
| 2: Methods | Provide a clear description of the methods used for PPI in the study | 6, 8 |
| 3: Study results | Outcomes—Report the results of PPI in the study, including both positive and negative outcomes | 9 |
| 4: Discussion and conclusions | Outcomes—Comment on the extent to which PPI influenced the study overall. Describe positive and negative effects | 15 |
| 5: Reflections/critical perspective | Comment critically on the study, reflecting on the things that went well and those that did not, so others can learn from this experience | 19 |
