# Supplementary material for: Co-producing an online patient public community research hub: a qualitative study exploring the perspectives of national institute for health research (NIHR) research champions in England
Source: Res Involv Engagem. 2024 Feb 16;10:26. doi: 10.1186/s40900-024-00556-4 (PMC10874083; doi:10.1186/s40900-024-00556-4)
Supplement: Supplementary file 2 — Additional file 2. Plain Language Summary. [file 40900_2024_556_MOESM2_ESM.docx]

**Plain Language Summary**

**Aim(s) of the research**

The aims of this research were to find out what research volunteers within the National Institute for Health Research (NIHR), in the UK, would like to find on a university website about health and care research. This research aimed to understand how best to raise awareness about how people can get involved in research. It also aimed to understand how best to share information about research, with patients and members of the public, from a university website.

**How might patients and members of the public and services benefit from this research?**

This research may help to improve how the results of research studies get to patients and members of the public. This is a part of research that is often forgotten about by researchers. It may also help to provide a place for researchers to share opportunities for patients and members of the public to find out about ways to become involved in research.

This research has written recommendations that have been co-produced with NIHR Research Champions. The recommendations are for researchers, other organisations, or services to use. They aim to help to improve how information gets shared about the results of research and ways in which patients and members of the public can get involved.

**Background to the research**

Universities have an important role to play in providing opportunities for patients and members of the public to be involved in the development of research studies. Researchers working in universities should share the results of their studies with patients and members of the public, but this does not always happen. It is important to patients and members of the public that they are informed of the results of studies. It should also be easy for patients and members of the public to find out about opportunities to get involved with researchers in the development of their research. This should include making an online place for patients and members of the public to find this information, but there has not been much research done about how best to do this.

**Design and methods used**

*Patient Public Involvement*

NIHR Research Champions were involved in the co-production of the project. This means that shaping the research was shared between the researcher and the NIHR Research Champions. NIHR Research Champions are patients and members of the public who often have experience of taking part in research and so have some understanding about how it works.

Five NIHR Research Champions from diverse ethnic and cultural backgrounds (including younger and older people) helped to develop a set of webpages on a university website, called the Patient Public Community Research Hub. The group were involved in deciding what was important to include on the hub, as well as developing a set of recommendations.

*Data collection*

Once the initial online hub was created, online interviews were held with other NIHR Research Champions. The interviews were to help the researcher to understand what they thought about the Patient Public Community Research Hub. There were 15 NIHR Research Champions who took part in two interviews each. The first interview was a “walk through” , or an opportunity to say out loud what NIHR Research Champions thought about the hub. The second interview which occurred approximately one week later, was to ask more in-depth questions about what they thought about the hub.

The results from the interviews were analysed and grouped into themes. The themes helped to tell us what NIHR Research Champions felt patients and members of the public would want to see on the Patient Public Community Research Hub and what areas needed improving.

**Dissemination**

The results of this research will be shared through a peer-reviewed scientific journal. They will also be shared as a Plain Language Summary on the Patient Public Community Research Hub at Warwick Medical School. Results will be shared more widely with the NIHR, researchers and other organisations that are involved in the development of health and care research.
